# Comparative genomics reveals the high diversity and adaptation strategies of *Polaromonas* from polar environments

**DOI:** 10.1186/s12864-025-11410-6

**Published:** 2025-03-14

**Authors:** Yuntong Du, Changhua He, Karen G. Lloyd, Tatiana A. Vishnivetskaya, Hongpeng Cui, Bing Li, Da Gong, Xiaopeng Fan, Dayi Zhang, Hongchen Jiang, Renxing Liang

**Affiliations:** 1 State Key Laboratory of Geomicrobiology and Environmental Changes, China University of Geosciences, Wuhan, 430074 China; 2 Department of Microbiology, University of Tennessee, Knoxville, TN USA; 3 School of Ocean Sciences, China University of Geosciences (Beijing), Beijing, 100083 China; 4 School of Engineering and Technology, China University of Geosciences, Beijing, 100083 China; 5 Polar Research Centre, Jilin University, Changchun, Jilin 130061 China

**Keywords:** *Polaromonas*, Evolutionary history, Genomic adaptation, Comparative genomics, Carbon cycling

## Abstract

**Background:**

Bacteria from the genus *Polaromonas* are dominant phylotypes found in a variety of low-temperature environments in polar regions. The diversity and biogeographic distribution of *Polaromonas* have been largely expanded on the basis of 16 S rRNA gene amplicon sequencing. However, the evolution and cold adaptation mechanisms of *Polaromonas* from polar regions are poorly understood at the genomic level.

**Results:**

A total of 202 genomes of the genus *Polaromonas* were analyzed, and 121 different species were delineated on the basis of average nucleotide identity (ANI) and phylogenomic placements. Remarkably, 8 genomes recovered from polar environments clustered into a separate clade (‘polar group’ hereafter). The genome size, coding density and coding sequences (CDSs) of the polar group were significantly different from those of other nonpolar *Polaromonas*. Furthermore, the enrichment of genes involved in carbohydrate and peptide metabolism was evident in the polar group. In addition, genes encoding proteins related to betaine synthesis and transport were increased in the genomes from the polar group. Phylogenomic analysis revealed that two different evolutionary scenarios may explain the adaptation of *Polaromonas* to cold environments in polar regions.

**Conclusions:**

The global distribution of the genus *Polaromonas* highlights its strong adaptability in both polar and nonpolar environments. Species delineation significantly expands our understanding of the diversity of the *Polaromonas* genus on a global scale. In this study, a polar-specific clade was found, which may represent a specific ecotype well adapted to polar environments. Collectively, genomic insight into the metabolic diversity, evolution and adaptation of the genus *Polaromonas* at the genome level provides a genetic basis for understanding the potential response mechanisms of *Polaromonas* to global warming in polar regions.

**Supplementary Information:**

The online version contains supplementary material available at 10.1186/s12864-025-11410-6.

## Background

Environments with temperatures below 5^°^C year-round are generally considered permanently cold environments [1]. 75% of the Earth’s biosphere is located in permanently cold regions (e.g., Antarctica, the Arctic, high mountain areas, the deep sea, ice caves and the upper atmosphere) [1, 2]. These low-temperature environments impose significant stresses (freezing, desiccation and high osmolarity) on the survival of microorganisms [3, 4]. Despite the harsh conditions in cold environments, cryophilic microorganisms exist and remain metabolically active even at the ambient temperatures typical of these regions [5–8]. These cold-loving microorganisms have developed unique adaptation strategies [9–12] that allow them to participate in various metabolic processes and contribute to global biogeochemical cycles in cryospheric ecosystems [13–15].

Among the known psychrophilic microorganisms, bacteria of the genus *Polaromonas* are the dominant phylotypes from polar and high-elevation environments [16, 17]. Pure culture experiments have confirmed that most strains of *Polaromonas* are heterotrophic microorganisms capable of degrading organic carbon [18–20]. However, several strains of *Polaromonas* also possess the ability to grow chemolithotrophically on H_2_:CO_2_, such as *Polaromonas hydrogenivorans* and *Polaromonas naphthalenivorans* [21]. In addition, metagenomic insights into diazotrophic communities across Arctic glacial forefields revealed that *Polaromonas* is also a key nitrogen-fixing microorganism [22]. Global warming is causing glaciers to retreat and permafrost to melt, causing the cryosphere to shrink on a global scale [23, 24]. This phenomenon also affects the activity of microorganisms in cold environments. For instance, thawing permafrost leads to increased microbial activity and increased emissions of greenhouse gases such as CO_2_, CH_4_ and N_2_O [25]. However, the response of microbes to climate warming in cold environments is still not fully understood. As chemoheterotrophic microorganisms that can utilize a variety of organic carbon sources to produce CO_2_, *Polaromonas* plays an important role in the carbon cycle in the cryosphere ecosystems. It is reasonable to hypothesize *Polaromonas* in polar regions will respond to global warming and may produce more greenhouse gases to create a positive feedback effect on climate change. A comprehensive understanding of metabolic function and adaptation strategies at the genomic level can provide a genetic basis for predicting the responses of *Polaromonas* to global climate change in polar glacial and permafrost ecosystems.

The genus *Polaromonas* was first proposed by Irgens et al. (1996) to describe the psychrophilic marine bacterium *Polaromonas vacuolata* isolated from Antarctica [18]. Subsequently, *Polaromonas* strains were successfully isolated and cultured from various sediments in polar and nonpolar regions [20, 21, 26–29]. On the basis of 16 S rRNA gene amplicon sequences and a polyphasic approach, their taxonomic positions were clarified. However, microbial pure culture research is unable to cover the full diversity of *Polaramonas* strains in the environment because of its relatively slow throughput. Owing to the lack of sufficient analysis of *Polaromonas* genomes at a global scale, our understanding of the ecological role and potential applications of *Polaromonas* is still limited. With the rapid development of high-throughput sequencing technology [30], the turnover time and cost of metagenomic sequencing have decreased greatly. The widespread application of genome-centered metagenomics in recent years has enabled the discovery of numerous draft genomes (metagenome assembled genomes, MAGs) from previously uncultured *Polaromonas*. Comparative genomic studies can provide comprehensive information about the metabolism, evolution and speciation of microorganisms [31–33]. In particular, genomic comparisons of species within the same genus can shed light on how environmental conditions affect the extent of genomic diversity and evolution of different species. Therefore, we conducted a comparative genomic analysis with the genomic data at NCBI to understand the evolution and adaptation of *Polaromonas* in cold environments.

In the present study, 202 *Polaromonas* genomes were retrieved from public databases and examined for phylogenetic diversity. A unique ecological clade from the polar region was identified in the phylogenetic tree. A comparative genomic analysis of this clade and a reference group from nonpolar regions revealed that *Polaromonas* species from polar regions had larger genome sizes with greater gene density. Furthermore, we observed two distinct evolutionary patterns in *Polaromonas* in this clade containing only genomes from polar environments. Betaine was found to be crucial for the cold adaptation of *Polaromonas*. Our findings significantly improve our understanding of the metabolic capabilities, habitat adaptations, and evolutionary processes of *Polaromonas* in polar and high-altitude ecosystems.

## Materials and methods

### Acquisition of *Polaromonas* genomic data

A total of 249 genomes initially classified as *Polaromonas* were retrieved from the NCBI Genome Assembly database (update to September 2023). To verify the taxonomy assignment in NCBI, all downloaded genomes were re-classified using the workflow implemented in GTDB-Tk v2.3.2 [34]. The preliminary reclassification suggested that only 202 of the 249 genomes belonged to the genus *Polaromonas*. Therefore, the 47 genomes belonging to other genera, including *Hydrogenophaga*,* Rhodoferax* and *JAAFIP01*, were excluded from this study. The quality of all the genomes was assessed using CheckM v2-1.0.2 with the default options [35]. A total of 86 high-quality genomes (> 90% completeness) were selected for downstream analyses.

### Phylogenomic analysis

Pangenome phylogenetic analysis was conducted on the basis of concatenated amino acid sequences. A total of 120 up-to-date conserved marker genes were identified by the software GTDB-Tk v2.3.2 with its pipeline and default parameters ( [34]. The marker genes from all the genomes were subsequently aligned with HMMER v3.3.2 [36], and the phylogenomic trees were subsequently reconstructed on the basis of concatenated alignment of 120 conserved marker genes by the IQ-TREE v2.2.2.7 with the maximum likelihood (ML) method under the LG + I + G protein substitution model ( [37]. Bootstrap analysis with 1,000 replicates was performed to determine the reliability of the branches obtained from the two phylogenomic trees ( [38]. Genomes from *Rhodoferax* and *JAAFIR01* were chosen as the outgroup to root the tree. The subsequent editing of the phylogenetic tree was completed using the ITOL tool ( [39] (https://itol.embl.de/). The pairwise average nucleotide identity (ANI) analysis of all the genomes was performed using fastANI-v1.33 ( [40].

### Functional annotation

Functional annotation of genomes was determined by comparing the predicted genes against the NCBI nonredundant database (nr), Kyoto Encyclopedia of Genes and Genomes (KEGG) ( [41], Orthologous Genes (COG) ( [42], and Pfam protein families (Pfam) databases ( [43] using MMseqs v0.0.1 ( [44] with E-value of < 1e-^15^. Carbohydrate-active enzymes were specifically annotated using the dbCAN ( [45] database alongside HMMER v3.3.2 ( [36] with coverage of 0.35 and E value of 1e-^15^ providing a comprehensive understanding of the metabolic potential for carbohydrate utilization. Additionally, peptidase annotation was conducted using the MEROPS database ( [46] along with MMseqs data ( [44] with coverage of 0.4 and E value of 1e^− 15^.

### Comparative genomics analysis

A total of 16 genomes of close relatives from nonpolar regions were selected as the reference group (i.e., the ‘nonpolar group’). Comparative genomic analyses were performed in both groups to investigate the genomic features and mechanisms of adaptation to extremely cold environments. The genome size, G + C content, CDSs and coding density of the genomes were annotated by the Bakta workflow and compared between the two groups using the Mann–Whitney test by Spssau ( [47] (https://spssau.com/About_spssau.html). On the basis of the results of the functional annotations, the genes related to carbohydrate and peptide metabolism in the polar and nonpolar groups were compared. In addition, osmotic genes related to compatible solute biosynthesis and transport were compared between the two groups.

### Statistical analysis

The pangenome data were visualized using the base R graphics unless otherwise specified. R v4.1.3 and ggplot2 v3.4.4 were used for statistical analysis and plotting. The following R packages were used for mapping strain isolation sites: ggmap v4.0.0, maptools v1.1–6, maps v3.4.1, and sp version 1.6–0. Heatmaps of protein-related genes were obtained by the pheatmap package in R. Box plots were drawn with ggplot2 v3.4.4.

## Results

### Global distribution of *Polaromonas*

**Fig. 1:**
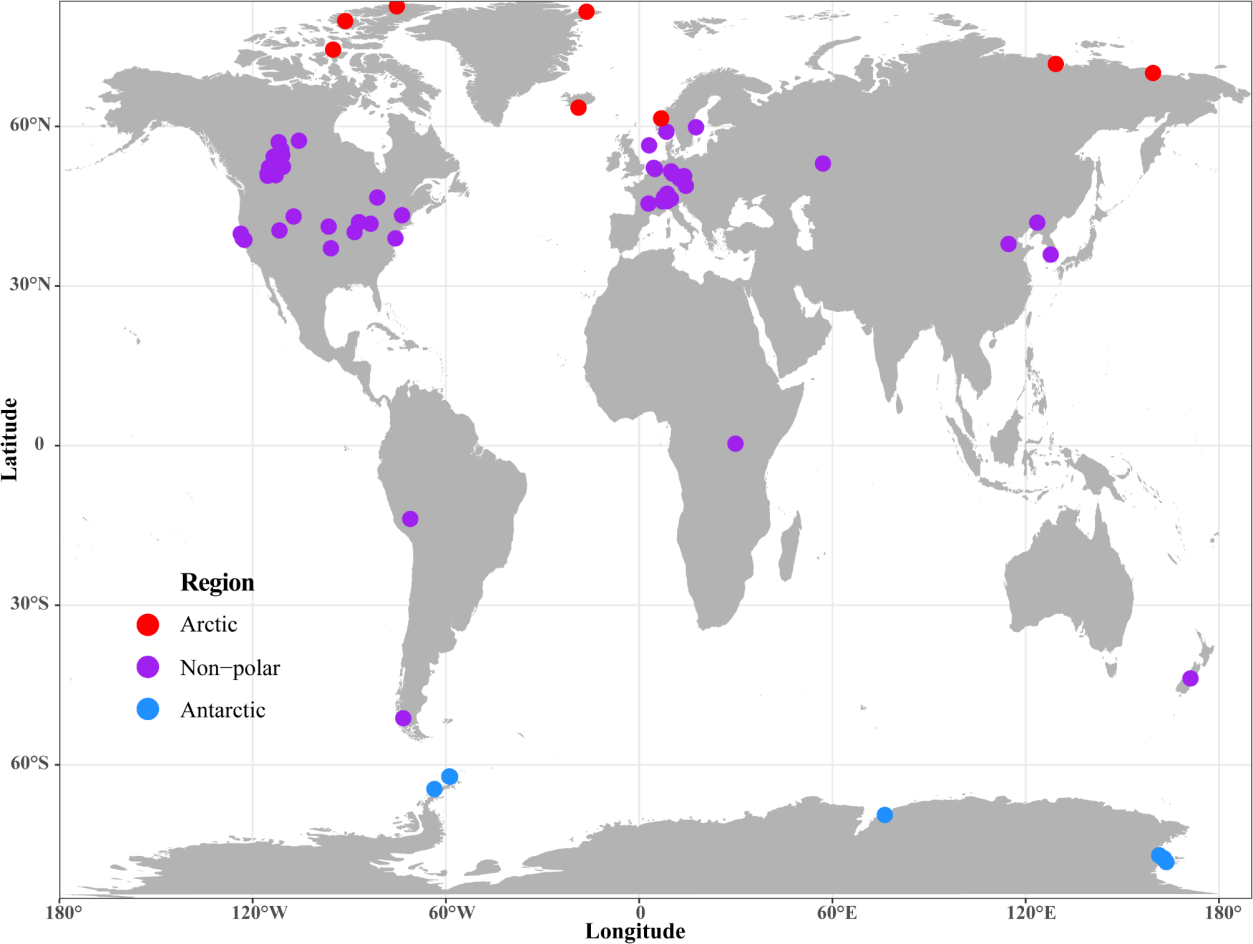
Locations of worldwide sampling sites of *Polaromonas* on 6 continents. The colors correspond to different regions, with red for the Arctic, purple for the nonpolar regions and blue for the Antarctic

Among the 202 genomes, 176 were metagenome-assembled genomes (MAGs) recovered from the environment, whereas the remaining 26 were whole genomes sequenced from pure cultures (Table S1). The global distribution of *Polaromonas* across different habitats is depicted in Fig. 1. *Polaromonas* spp. have been retrieved from various cold environments globally, including Anvers Island in Antarctica (GCF_012584515) and the Norway glacial foreland till (GCA_000709345) (Fig. 1, Table S1). According to the latitude and longitude information (Table S1), 29 of the 202 genomes originated from high-latitude regions, including 14 genomes from Antarctica and 15 genomes from the Arctic. The remaining 166 genomes were from nonpolar regions, which included 11 genomes from low-latitude areas. More detailed genome information is provided in the supplementary material (Table S1).

### Taxonomy and phylogeny of *Polaromonas*

**Fig. 2:**
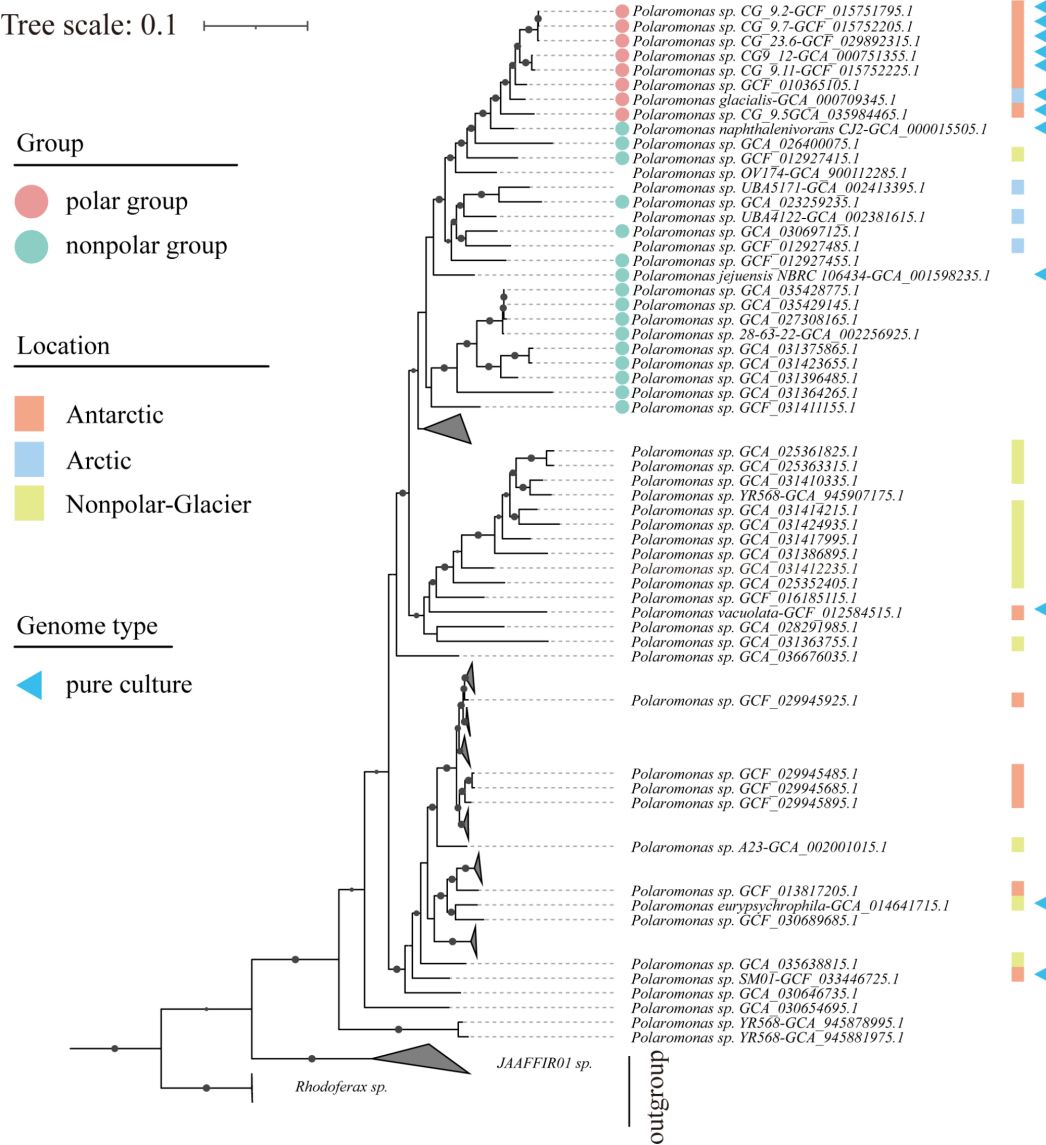
Phylogenetic tree constructed with 86 high-quality genomes (completeness > 90%) of *Polaromonas* and 3 genomes from *Rhodoferax* and 4 genomes from *JAAFIR01* (the outgroup). The tree was reconstructed using the maximum likelihood algorithm on the basis of 120 conserved marker genes. The polar group was defined as comprising 8 *Polaromonas* genomes from polar environments, all of which clustered within the same clade. The scale bar represents 0.1 amino acid substitutions per site

**Fig. 3:**
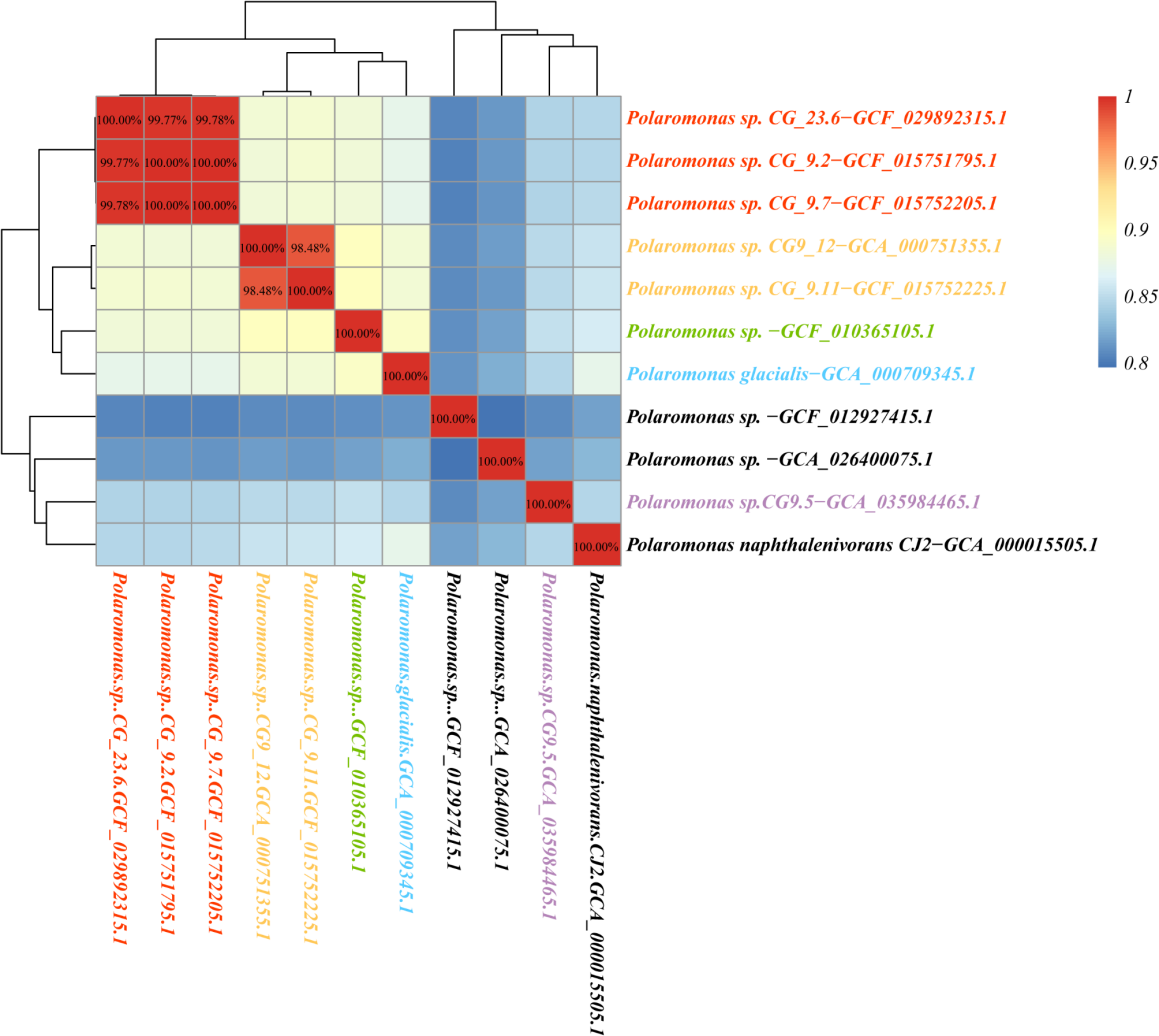
Heatmap showing ANI analysis of 8 genomes from the polar group and 3 closely related genomes from the nonpolar region. The ANI values greater than 95% are shown in the figure. Various species are distinguished by color in the figure, and the labels for nonpolar genomes are black

All *Polaromonas* genomes from around the world form a monophyletic clade (Figure S1) that is distant from its closest sister clade (*Rhodoferax)*. The *Polaromonas* clade encompasses four well-characterized strains of *Polaromonas*, validating the taxonomic identity of all the *Polaromonas* genomes for the entire clade. On the basis of phylogenetic placements and ANI values for species delineation (95-96% cutoff) ( [48], the 202 *Polaromonas* genomes were reclassified into 121 distinct species (Figure S1). Further phylogenomic analysis of high-quality genomes (86 genomes) revealed that 8 genomes originating from polar regions (*Polaromonas sp. CG_9.2-GCF_015751795.1*,* Polaromonas sp. CG_9.7-GCF_015752205.1*,* Polaromonas sp. CG_23.6-GCF_029892315.1*,* Polaromonas sp. CG9_12-GCA_000751355.1*,* Polaromonas sp. CG_9.11-GCF_015752225.1*,* Polaromonas sp. GCF_010365105.1*,* Polaromonas glacialis -GCA_000709345.1* and *Polaromonas sp CG_9.5-GCA_035984465*) cluster into a unique clade (Fig. 2), whereas the remaining polar genomes are dispersed throughout the phylogenetic tree. In addition, this clade was closer to modern species than other genomes in the tree. Among these 8 genomes, 3 genomes *(Polaromonas sp. CG_9.2-GCF_015751795.1*, *Polaromonas sp*. *CG_9.7-GCF_015752205.1* and *Polaromonas sp*. *CG_23.6-GCF_029892315.1)* were identified as the same species as the ANI values exceeded 99% (Fig. 3). Moreover, the other two genomes, *Polaromonas sp*. *CG9_12-GCA_000751355.1* and *Polaromonas sp*. *CG_9.11-GCF_015752225.1*, were confirmed to be the same species on the basis of the ANI threshold (95-96%) ( [48]. *Polaromonas sp*. *GCF_010365105.1*, *Polaromonas glacialis -GCA_000709345.1* and *Polaromonas sp CG_9.5-GCA_035984465* were identified as three separate species on the basis of their relatively low ANI values (< 95%). Ultimately, five distinct species were delineated among the 8 genomes classified into the polar group (Figs. 2 and 3).

### Transmission and evolutionary history of *Polaromonas*

**Fig. 4:**
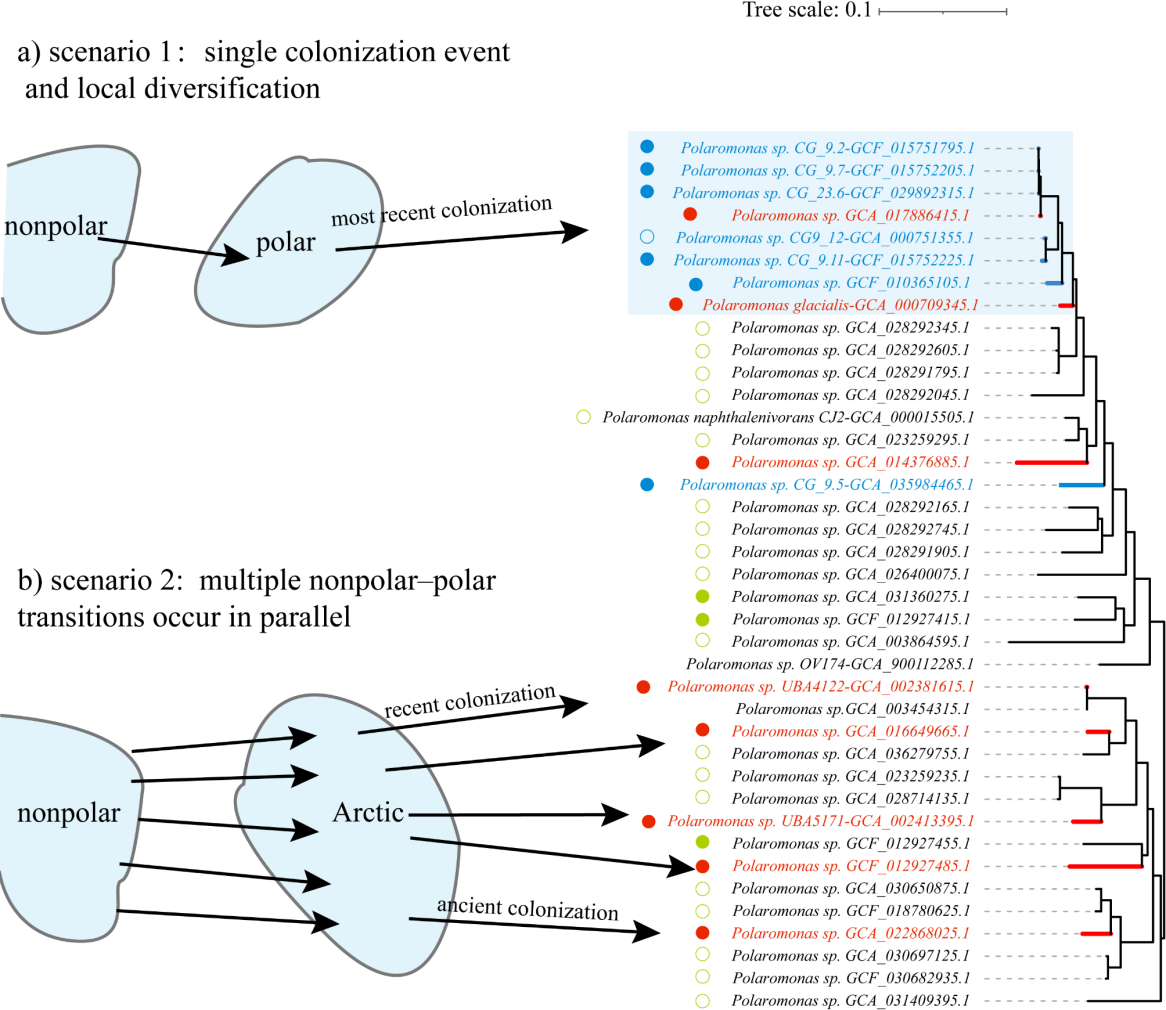
Phylogenies and inference of historical nonpolar–polar transitions of *Polaromonas*. The tree was constructed by the maximum likelihood method with 202 *Polaromonas* genomes. A partial phylogenetic tree from Figure S1 was intercepted to illustrate the transmission and evolutionary relationships of *Polaromonas.* The black lines represent the nonpolar genomes, whereas the red and blue lines represent the Arctic and Antarctica genomes, respectively. Additionally, the blue, red and green solid circles refer to genomes from Antarctic glaciers, Arctic glaciers and non-polar glacier, respectively. (**a**) Genomes from the polar regions cluster together and form a monophyletic clade, suggesting that these polar species spreaded to polar regions from nonpolar regions during a single colonization event and that diversification occurred later. (**b**) The transmission of *Polaromonas* to the Arctic from nonpolar regions occurred in parallel during the diversification of the analyzed lineages

Phylogenomic analyses of 202 *Polaromonas* genomes based on 120 conserved marker genes revealed two possible evolutionary scenarios for the diversification of polar and nonpolar species within the genus *Polaromonas* (Fig. 4). In the first possible scenario, 8 genomes from the polar regions clustered together as the most recent lineage at the top of the phylogenetic tree (Fig. 4a). This monophyletic clade included 6 genomes isolated from the Antarctic region and 2 genomes from the Arctic region. The polar group was most closely related to the other 3 genomes from nonpolar regions (Fig. 4, Table S1). In the second scenario, genomes derived mostly from the Arctic were phylogenetically dispersed within nonpolar lineages. For example, the Arctic species of a recent colonizer (*Polaromonas sp. UBA4122–GCA_002381615.1*) and *Polaromonas sp. GCA_003454315.1* from the nonpolar region converged to form a shared clade within the phylogenetic tree (Fig. 4b). Furthermore, *Polaromonas sp. GCF_012927485* from the Arctic and *Polaromonas sp. GCF_012927455.1* from the nonpolar region were clustered within another clade (Fig. 4b), suggesting that more ancient colonization events occurred for this species.

### Genomic comparison of *Polaromonas* from polar and nonpolar regions

**Fig. 5:**
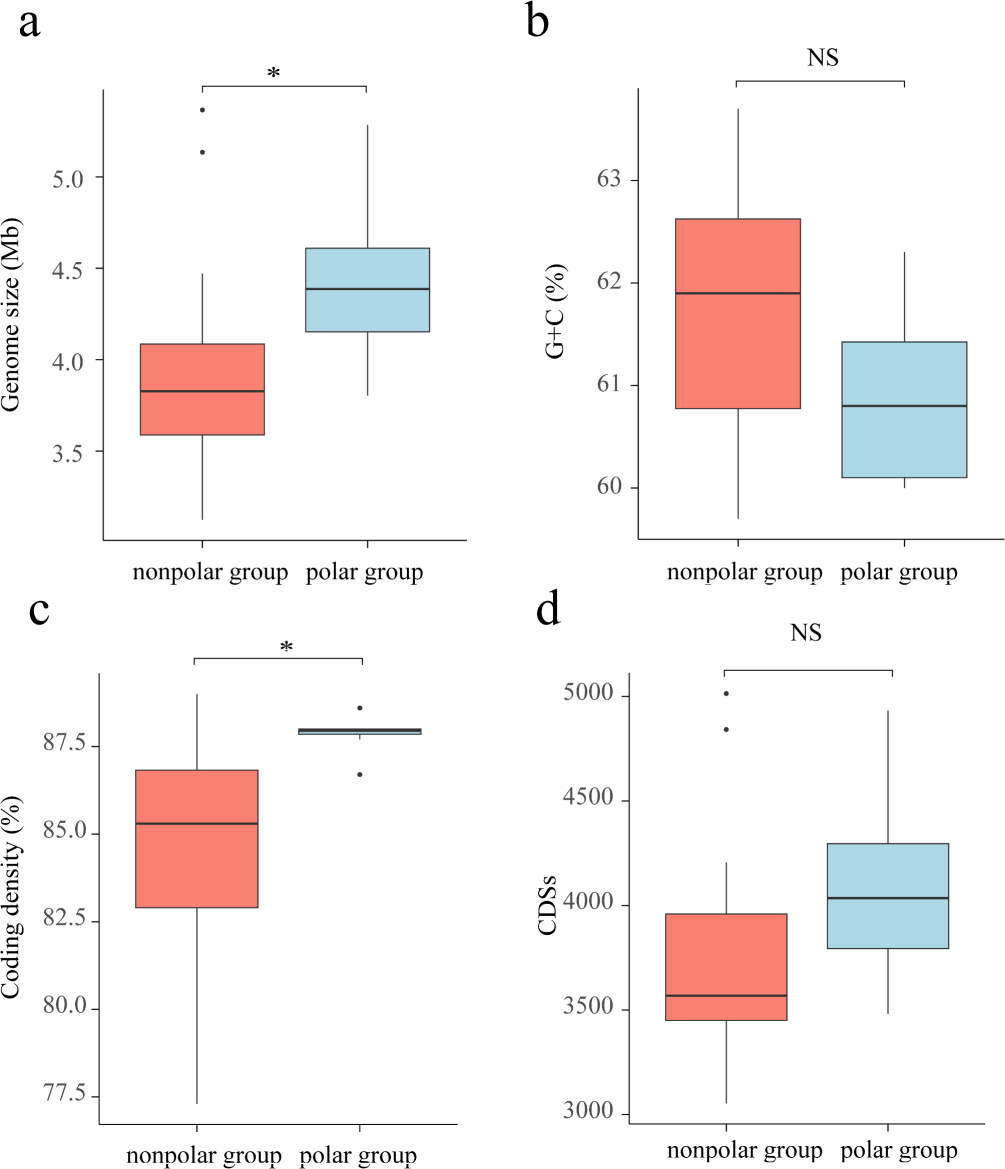
Comparison of the (**a**) genome size, (**b**) G + C content, (**c**) coding density, and (**d**) coding DNA sequences between the polar and nonpolar groups. Compared with the nonpolar group, the polar group presented a larger genome sizes and greater coding density; no significant difference in G + C content and CDSs was found (Mann‒Whitney test, *, *p* < 0.05; **, *p* < 0.01; NS, not significant)

Among the 8 genomes from polar regions, the genome size ranged from 4.15 Mb to 5.28 Mb, with an average size of approximately 4.45 Mb. The genomic G + C content varied from 60.00 to 62.30%, with an average of 60.88% (Table S2). The sizes of the closely related nonpolar genomes (16 high-quality genomes, ‘nonpolar group’) ranged from 3.12 Mb to 5.37 Mb, with an average of 3.94 Mb. The genomic G + C content ranged from 59.70 to 63.70%, with an average of 61.65% (Table S2). A comparison of the G + C content between the polar and nonpolar groups revealed no significant differences (Mann‒Whitney test; *p* = 0.198 > 0.05; Fig. 5b). However, the polar group presented a greater number of genome sizes and higher coding density, indicating a denser packing of genes (Mann‒Whitney test; *p* = 0.017–0.023 < 0.05; Fig. 5). This increased coding density resulted in a greater number of carbon metabolic pathways, with an average of 116 pathways in the polar group compared with 107 pathways in the nonpolar group. Statistical analysis confirmed the significance of this difference (Mann‒Whitney test; *p* = 0.023–0.034 < 0.05; p3 = 0.623 > 0.05; Figure S2).

### Metabolic functions of *Polaromonas*

**Fig. 6:**
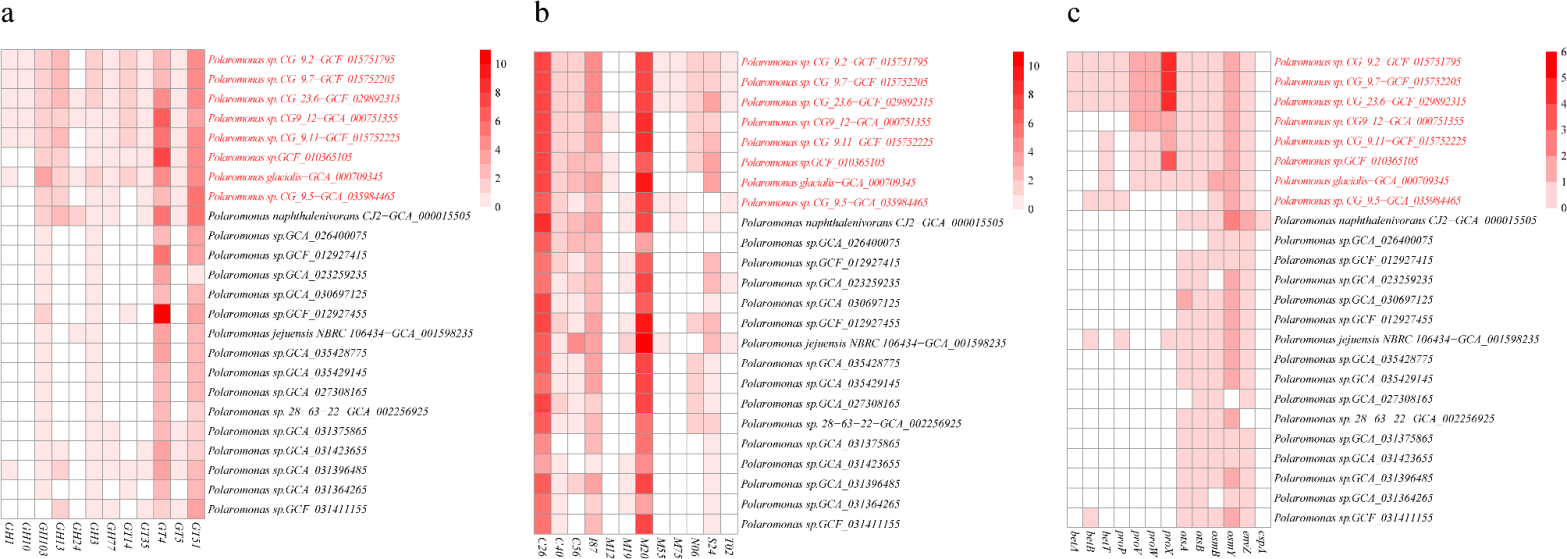
Heatmap showing the enrichment of predicted genes encoding carbohydrate-active enzymes (**a**), peptidases (**b**) and cold adaptation genes (**c**). The 8 genomes from the polar group are highlighted in red. The color scale represents the number of genes identified in the genomes

All *Polaromonas* genomes harbor conserved central carbon metabolism pathways, including glycolysis, the tricarboxylic acid cycle, the pentose phosphate pathway and glyoxylate metabolism (Table S6). Notably, pathways for galactose, fructose, mannose, starch and sucrose metabolism were present in all the genomes, suggesting that these sugar compounds could serve as carbon sources for *Polaromonas* (Table S6). The *Polaromonas* genomes contain several genes encoding glycosyltransferases (*GTs*) and glycoside hydrolases (*GHs*), with *GTs* being the most abundant (Fig. 6a). Among the analyzed genomes, those originating from polar regions (GCA_000709345, GCF_010365105, and GCA_000751355) had the greatest number (78) of genes encoding carbohydrate-active enzymes (CAZymes) (Table S4). The glycosyltransferases identified as various types (*GT4 and GT51*) were the most prevalent (Fig. 6a). Statistical analysis revealed a significant difference in the abundance of genes encoding *GT51*, with an average of 5 in polar genomes compared with 3.15 in nonpolar genomes. Additionally, two glycoside hydrolases, *GH103* and *GH3*, were enriched in polar genomes (Fig. 6a). Furthermore, genes related to peptide metabolism were abundant in most genomes in the polar group (Mann‒Whitney test, *p* = 0.002–0.01 < 0.05) (Fig. 6b). For example, all genomes from the genus *Polaromonas* harbor homologous genes involved in ammonium metabolism (*C26*,* C40 family*) (Fig. 6b, Table S4), and more copies of these genes are in the polar genomes (Mann‒Whitney test, *p* = 0.002–0.01 < 0.05).

### Adaptation to extremely cold conditions

Several cold adaptation genes differed greatly between the polar and nonpolar *Polaromonas* groups (Fig. 6c). A significant increase in the abundance of genes associated with betaine synthesis (*betA*,* betB*) and transport (*proX*,* proW*,* proV*) was detected in the polar genomes compared with the nonpolar ones (Mann–Whitney test, *p* < 0.01) (Fig. 6c). However, other cold stress genes did not differ between the polar and nonpolar *Polaromonas* groups. Genes involved in trehalose biosynthesis (*otsA* and *otsB)* were identified across most *Polaromonas* genomes (Fig. 6c), but the genes associated with the transport of trehalose (*thuG*,* thuF*,* thuE*) were all absent in the analyzed genomes (Table S3). In addition, there was no significant difference in trehalose synthesis genes (*otsA* and *otsB*) between the two groups (Fig. 6c, Table S3). The genes encoding universal stress proteins (*UspA family*) are present in all *Polaromonas* genomes, and 9 copies of the *UspA* gene were identified in GCA_000709345 (isolated from Norway) (Table S3). However, the Mann‒Whitney test revealed that the number of copies of the *UspA* gene between the polar and nonpolar groups was not significantly different. Interestingly, genomic analysis of *Polaromonas* revealed that genes encoding cold shock proteins (*cspA*) were generally absent in the analyzed genomes (Fig. 6c). Only one copy of the cold shock protein-encoding gene was identified in the genome of GCA_000015505. Genes involved in regulating cell osmotic pressure (*Osm Y*,* Osm B*,* and envZ*) were present in most of the genomes, and no significant difference was detected between the polar and nonpolar groups (Fig. 6c).

## Discussion

The goal of this study was to explore the genomic diversity and cold adaptation strategies of *Polaromonas* on the basis of large-scale genomic datasets. Our study revealed the global distribution of *Polaromonas* phylotypes at the genomic level (Fig. 1, Table S1), which is consistent with the cosmopolitan distribution commonly observed in many prokaryotes at various geographic scales ( [49]. Members of *Polaromonas* have been isolated not only from polar glaciers and periglacial sediments ( [26, 27] but also from temperate soil ( [29, 50]. Previous studies illustrated the global distribution of *Polaromonas* phylotypes using 16 S rRNA gene amplicon sequencing ( [16, 17, 51]. Although taxonomic profiling can provide broader global distribution information, the advantage of genomic distribution is that it can provide more information on genomic and metabolic diversity, survival and adaptation mechanisms. Our analysis of 202 genomes (cultured strains and MAGs) provides further evidence for this widespread distribution of *Polaromonas*. The global distribution of *Polaromonas* phylotypes suggested that they have strong environmental adaptability. Previous genetic analysis based on the 16 S rRNA gene revealed that the genetic distance of *Polaromonas* increases with geographical distance ( [16, 51]. However, contrasting evidence indicates that sequences collected at widely separated sites (9400–13500 km) are more similar than those classes retrieved at other distances ( [51]. The latter finding was supported by our comprehensive genomic analysis. For example, genomes from the Antarctica and the Arctic or genomes from polar and nonpolar regions clustered together as the same species in the phylogenetic tree (Figure S1). Therefore, the distribution of *Polaromonas* phylotypes around the globe appears to be faster than species radiations in this genus, at least for some species. To date, 9 species have been proposed for pure cultures isolated from different environments ( [50]. The combination of average nucleotide identity (ANI) and phylogenomic analyses revealed that at least 121 species were delineated in our study (Figure S1), suggesting that the genetic diversity of cultured isolates may be expanded with further culturing efforts.

Two scenarios provide insights into the transmission and evolutionary histories of polar species as they continually adapt to changing environmental conditions in polar regions. In the first scenario, 8 polar genomes clustered together as a monophyletic group (Fig. 4a), suggesting that a single colonization event of *Polaromonas* in the polar region was followed by diversification within cold environments. It is plausible that continuous evolution of the first successful colonizer led to niche speciation within polar environments ( [17]. Previous studies on 16 S rRNA gene sequences suggested that biotic and abiotic factors may drive postselection niche separation and evolution within the *Polaromonas* genus ( [17]. Local speciation in cold environments aligns with this evolutionary trajectory (Fig. 4a), suggesting that the transmission of nonpolar *Polaromonas* to polar regions led to evolutionary divergence and an increase in species diversity in these cold environments. In the second scenario (Fig. 4b), polar and nonpolar species were closely related and dispersed across several lineages (Fig. 4b). In contrast to the first scenario, no distinction of exclusively polar clusters was observed in the phylogenetic tree (Fig. 4b). Multiple parallel colonization events suggested that nonpolar–polar transitions occurred repeatedly during the diversification of *Polaromonas* species. The long-distance transmission of *Polaromonas* from nonpolar to polar regions may be dependent on atmospheric transport ( [16]. The discovery of genomes associated with alpine air flow ( [52] has provided favorable evidence for this hypothesis. In addition, the *hipA* gene is considered an important factor that enables *Polaromonas* to remain dormant at high altitudes ( [16]. The multiple copies of the *hipA* genes discovered in the genomes provide further evidence for the airstream dispersal hypothesis (Table S3). Nevertheless, more work is needed to understand the transmission and evolutionary history of *Polaromonas* in cold environments.

Our results revealed an increase in genome size and the number of gene coding sequences in the polar genomes of *Polaromonas* (Fig. 5). Such genomic expansion could encode more genes and thereby *Polaromonas* with a broader range of mechanisms to address environmental challenges in extremely cold habitats in polar regions. Furthermore, reducing the G + C content of bacterial genomes promotes bacterial growth and DNA replication at relatively low temperatures [53]. *P. vacuolata* isolated from Antarctic sea ice adapts to cold in this way [54]. However, our comparative genomic analysis did not reveal this trend, since the G + C content of the polar group of *Polaromonas* genomes was not significantly different from that of the nonpolar group (60.88% vs. 61.65%, respectively). Therefore, we postulate that a reduction in G + C content may not be a universal adaptation mechanism to cold environments in the case of *Polaromonas* species.

A notable feature of psychrophiles is their enhanced ability to obtain energy and metabolize carbon at lower temperatures, which may allow for faster growth than at higher temperatures [55, 56]. This feature is well reflected in the *Polaromonas* genus. A larger genome size and more extensive genomic coding sequences correspond to an increase in carbon metabolic pathways in polar genomes (Figure S2). The increase in the abundance of genes related to glycoside hydrolases (*GHs*) and glycosyl transferases (*GTs*) in the polar genome suggests an improvement in carbohydrate metabolism in *Polaromonas* in extremely cold environments. Thus, *Polaromonas* might be able to use a wider range of carbon sources to generate energy in cold environments. In addition to being the source of carbohydrates, proteinaceous substrates can be hydrolyzed by *Polaromonas*, as shown by the presence of peptidases in most genomes (Fig. 6c). It appears that aspartic acid and hemipelagic acid seem to serve as nitrogen sources due to the presence of enzymes capable of breaking them down (Fig. 6b). The oligotrophic and low-temperature conditions of polar environments may require bacteria to develop more complex metabolic mechanisms than nonpolar moderate environments do. By using a broader range of carbon and nitrogen sources than nonpolar environments do, *Polaromonas* can produce enough energy to survive in low-temperature polar regions.

Studies of functional genes in the genome underscore the importance of betaine in the adaptation of *Polaromonas* to cold conditions. Enzymes such as cold shock proteins (*CSPs*) [55, 57, 58] and universal stress proteins (*UspAs*) ( [56] are pivotal in the response to environmental stressors. *CSPs* help maintain proper protein folding in cells at low temperatures, whereas *UspA* combats the oxidative stress that can be caused by extremely cold conditions. Nevertheless, our analysis revealed that there are few genes encoding *CSPs* in the polar genomes, indicating that these genes may not be crucial for the cold adaptation of *Polaromonas* (Fig. 6c). Conversely, our research revealed a significant number of *UspA* family genes in the *Polaromonas* genomes (Table S3), implying that *UspA* continues to provide protection against oxidative stress under cold conditions. In addition, psychrophilic bacteria are known to contain genes for the production of compatible solutes, such as trehalose and glycine betaine [59, 60], which help maintain osmotic pressure and stability at cold temperatures. The number of genes encoding the betaine synthesis pathway and betaine transporters is greater in *Polaromonas vacuolata* (isolated from beneath Antarctic Sea ice) than in other *Polaromonas* genomes [54]. In our study, genes associated with choline dehydrogenase and betaine transporters, which are involved in the initial phase of betaine biosynthesis [61], were more prevalent in polar genomes (Fig. 6c). These findings indicate that this clade may adapt to the increased osmotic pressure caused by cold conditions through the accumulation of betaine inside the cell.

## Conclusion

In summary, the number of *Polaromonas* species has dramatically increased by including all the complete genomes of pure cultures and MAGs of uncultured *Polaromonas* species in public databases. A monophyletic clade adapted to the cold polar environment was found in our study. Compared with the reference group from nonpolar regions, genomes with this clade have larger genome sizes and richer gene packing density. Furthermore, the polar clade genomes are enriched in functional genes. A greater number of genes involved in diverse carbohydrate metabolism and peptide metabolism, which are helpful in generating energy, are found in the polar group. Moreover, the importance of compatible solutes, e.g., betaine, in the regulation of microbial osmotic pressure in extremely cold environments has been further verified. Collectively, our results contribute to the broader knowledge of microbial life in polar regions and shed light on the unique strategies *Polaromonas* species employ to thrive in such harsh conditions. Owing to faster climate warming in polar regions, future research is desirable to better understand the responses of *Polaromonas* species and their adaptation to climate warming in cryosphere ecosystems such as glaciers and permafrost.

## Electronic supplementary material

Below is the link to the electronic supplementary material.

Supplementary Material 1

Supplementary Material 2

## Data Availability

All the genomic data were retrieved from public NCBI databases that are readily accessible (the accession numbers can be found in Table S1).
