# Supplementary material for: Comparative genomics reveals the high diversity and adaptation strategies of Polaromonas from polar environments
Source: BMC Genomics. 2025 Mar 14;26:248. doi: 10.1186/s12864-025-11410-6 (PMC11907789; doi:10.1186/s12864-025-11410-6)
Supplement: Supplementary file 2 — Supplementary Material 2 [file 12864_2025_11410_MOESM2_ESM.docx]

Comparative genomics reveals high diversity and adaptation strategies of *Polaromonas* from polar environments

Yuntong Du^1^, Changhua He^1^, Karen G. Lloyd^2^, Tatiana Vishnivetskaya^2^, Hongpeng Cui^3^, Bing Li^4^, Da Gong^5^, Xiaopeng Fan^5^, Dayi Zhang^5^, Hongchen Jiang^1^, Renxing Liang^1*^

1. State Key Laboratory of Geomicrobiology and Environmental Changes, China University of Geosciences, Wuhan, 430074, China
2. Department of Microbiology, University of Tennessee, Knoxville, TN, USA
3. School of Ocean Sciences, China University of Geosciences (Beijing), Beijing 100083, China
4. School of Engineering and Technology, China University of Geosciences, Beijing 100083, China
5. Polar Research Centre, Jilin University, Changchun 130061, Jilin, China

*For correspondence

E-mail: liangrenxing@cug.edu.cn

Mailing address: No. 68 Jincheng Street, East Lake High-tech Development Zone, Wuhan, Hubei Province, China, 430078

Tel: +86- 027-67883452


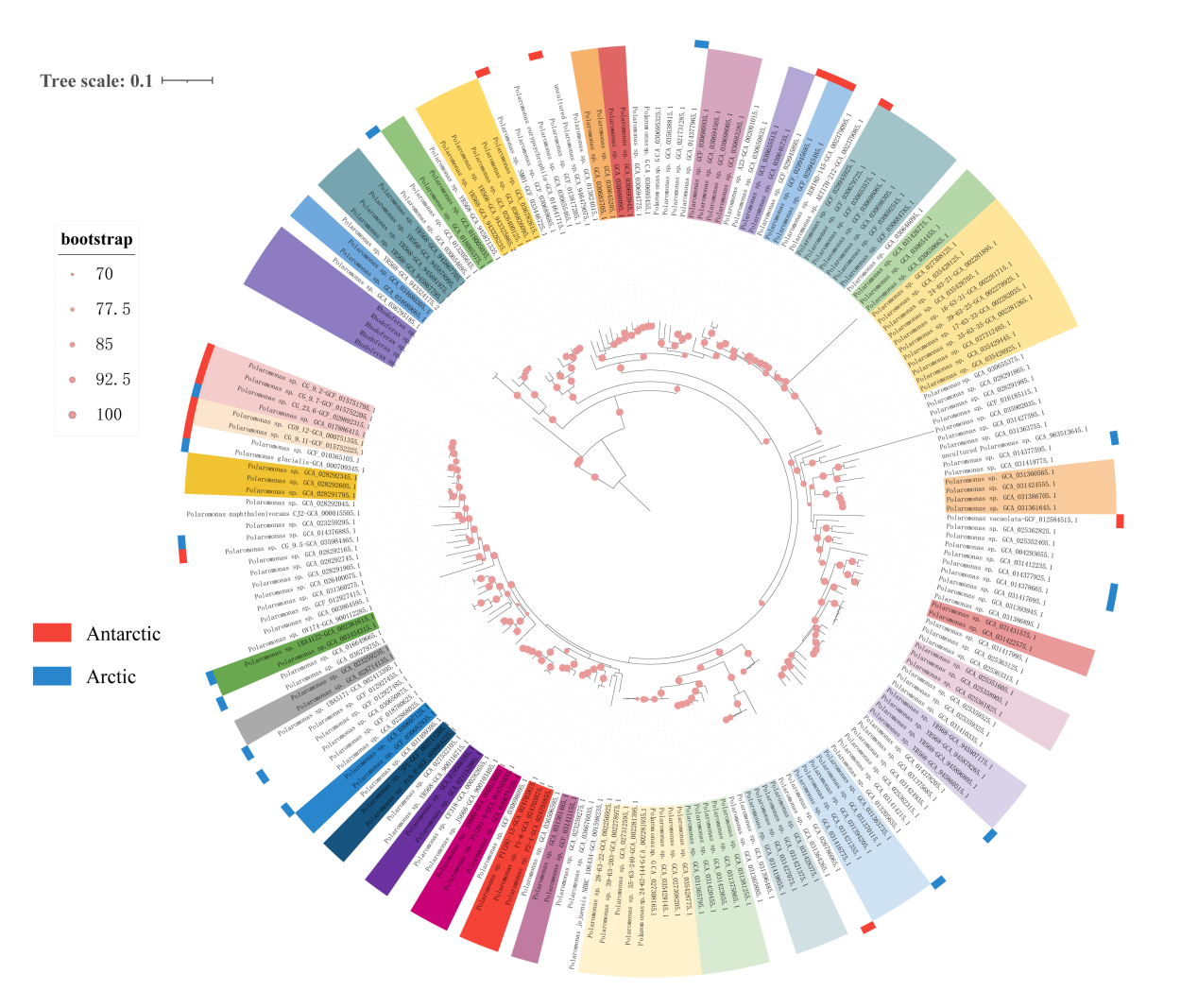


**Figure S1** *Polaromonas* phylogeny reconstructed using the maximum likelihood algorithm based on 120 conserved marker genes. 202 genomes from the genus *Polaromonas* are included and 5 genomes from *Rhodoferax* are selected as the outgroup. The background of different species were highlighted with distinct colors and thus the same background color indicates these genomes belonged to the same species. The outmost red bars indicate that the genomes are isolated from the Antarctic region whereas the blue ones represent the genomes from the Arctic region. The scale bar represents 0.1 amino acid substitution per site.


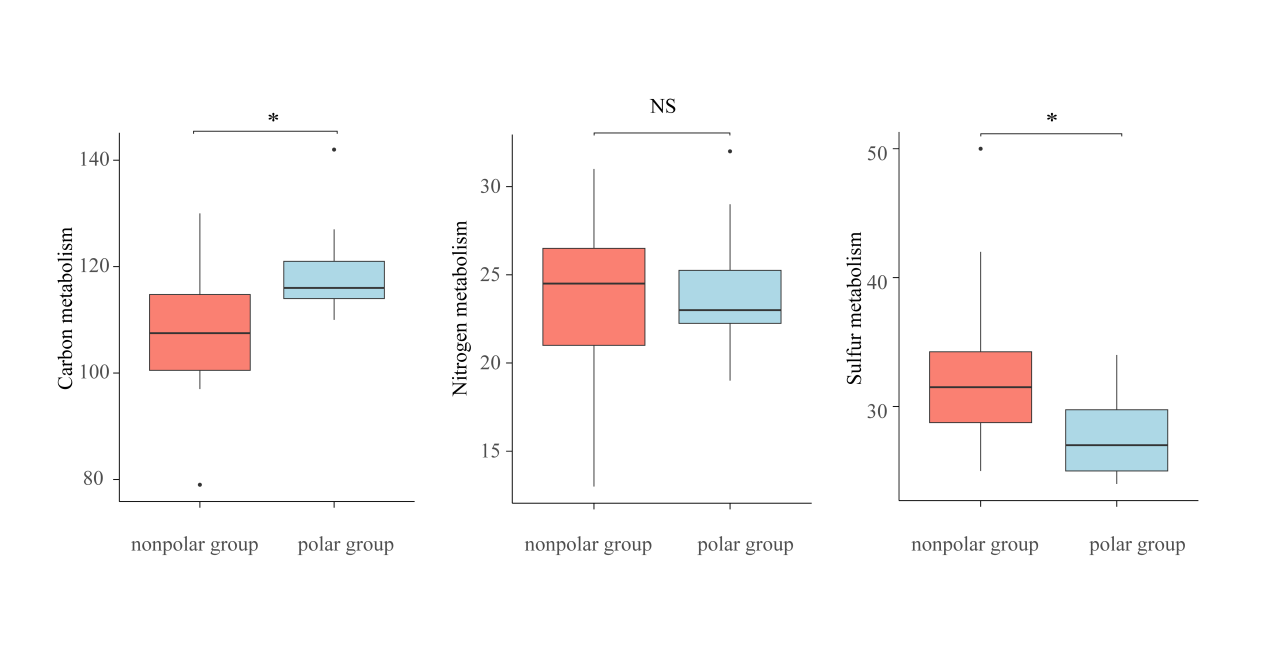


**Figure S2** Comparison of the (a) Carbon metabolism, (b) Nitrogen metabolism,(c) Sulfur metabolism pathways between the polar and nonpolar group. The polar group had a significantly higher number of Carbon metabolism pathway than the nonpolar group; no significant difference in G+C content was found (MannWhitney test, *, p<0.05; **, p<0.01; NS, not significant).


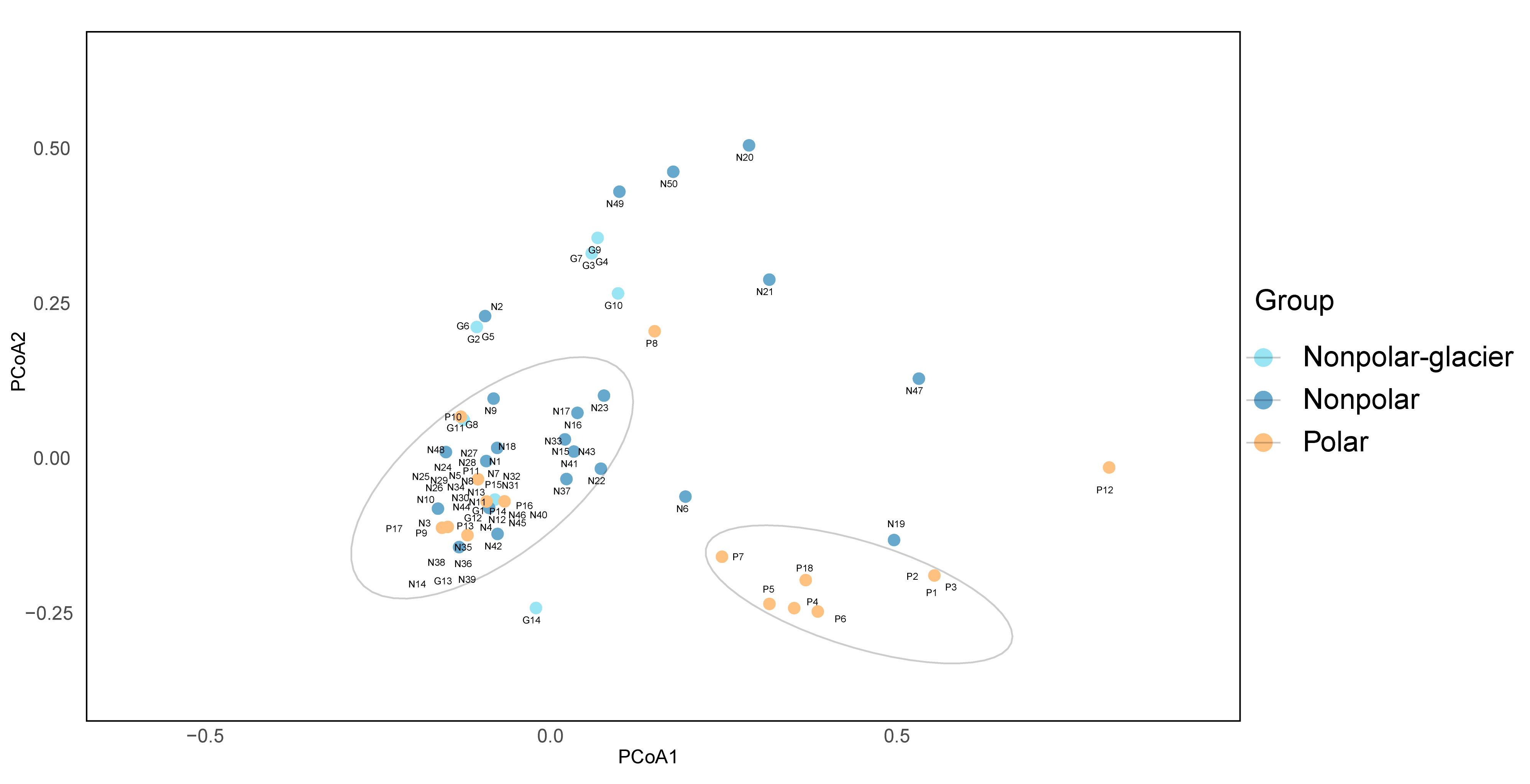


**Figure S3** The PCoA plot of genes related to cold adaption of Polar genomes,Nonpolar genomes and Nonpolar-glacier genomes. P1, 2, 3, 4, 5, 6, 7, 8 represented genomes that belonged to the independent clade in Figure 2.


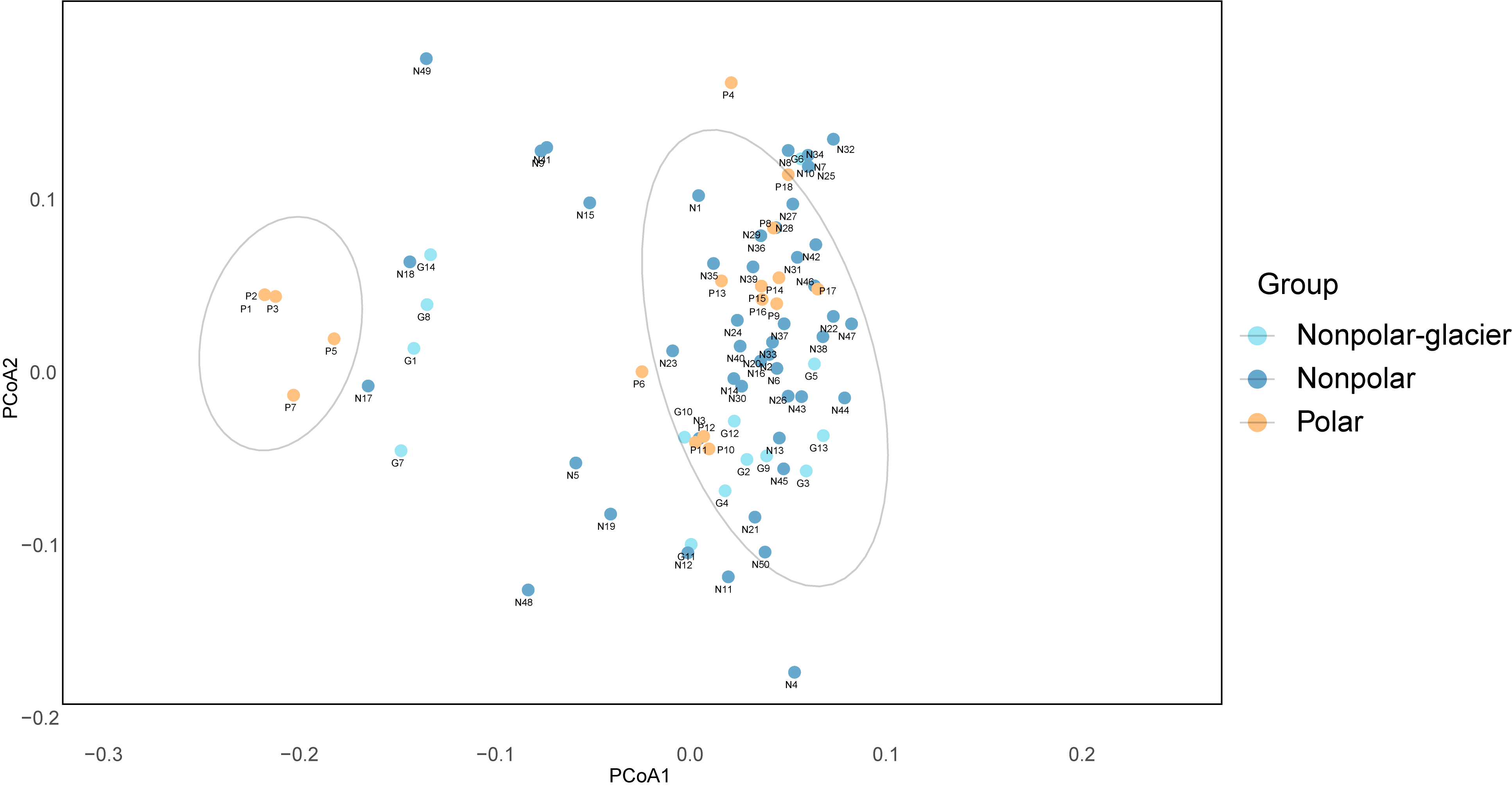


**Figure S4** The PCoA plot of genes related to carbohydrate and peptide metabolism of Polar genomes,Nonpolar genomes and Nonpolar-glacier genomes. P1, 2, 3, 4, 5, 6, 7, 8 represented genomes that belonged to the independent clade in Figure 2.
